# Public Healthcare: Citizen’s Preferences in Spain

**DOI:** 10.3390/healthcare8040467

**Published:** 2020-11-08

**Authors:** Silvia Prieto-Herraez, Teresa González-Arteaga, Rocío de Andrés Calle

**Affiliations:** 1 Foundations of Economic Analysis, University of Salamanca, E37007 Salamanca, Spain; silvi_ph@usal.es; 2 BORDA and PRESAD Research Groups, University of Valladolid, E47011 Valladolid, Spain; teresa.gonzalez.arteaga@uva.es; 3 BORDA Research Unit, PRESAD Research Group and Multidisciplinary Institute of Enterprise (IME), University of Salamanca, E37007 Salamanca, Spain

**Keywords:** healthcare barometer, preference stability, inter-temporal preferences

## Abstract

This paper analyzes the stability of citizens’ preferences on public healthcare services in Spain. Nowadays, the increasing privatization of some healthcare services and the rapid emergence of private hospitals have caused changes in people’s preferences on public healthcare systems. This paper focuses on analyzing the preferences of Spaniards on their healthcare system over time under the assumption that citizens’ preferences are represented by complete pre-orders. Data for this study were collected from the Spanish Health Barometer survey, and they were searched from 1995 until 2018. The results show that preferences on the public healthcare system are very stable along time.

## 1. Introduction

In recent years, there has been a change in the way public services in Spain are managed, especially healthcare services. Many healthcare services have been privatized, which has caused an open discussion in those countries where welfare state is an essential element. Pursuant to Kulesher and Forrestal [1], there are several kinds of healthcare financing models. Spanish healthcare system has undergone significant changes from 1967 until now, being in 1987 when the National Health System was created (see [2]). In particular, the National health system follows the *Beveridge model* that is distinguished by a universal care coverage of all citizens by central or regional governments. Alongside the public system, a private system has been growing in urban areas and it has grown quite substantially since the economic crises in 1990 and 2008. This proliferation was partly due to several facts. The elderly population growth and the reorganization and decentralization of the system are some of these factors in close connection with the high costs of public healthcare. Despite the fact that Spain spends 8.87% of its GDP per capita on healthcare according the most recent data available, many Spaniards consider that these funds could be allocated to healthcare more efficiently. Additionally, Spanish citizens present the second lowest degree of satisfaction with their healthcare system according to the latest public data [3].

Recently, researchers have shown an increased interest in studying how the preferences of citizens on healthcare systems have changed considering statistical and econometric modeling. Among others, the contributions of Martinussen and Magnussen [4] toward Norwegian National Health Service and Meleddu, Pulina, and Scuderi [5] Italian National Health Service can be highlighted.

One major theoretical issue that has dominated the field of *Decision-Making* for many years years has to do with the consideration of preferences as complete pre-orders in order to reflect the reality of citizens’ real situations (see [6,7], among other). However, the aforementioned studies are limited by the representation of preferences by numbers.

Taking into account these starting points, this paper focuses on analyzing the preferences of Spaniards on their healthcare system over time under the assumption that citizens’ preferences are represented by complete pre-orders. Therefore, the methodological approach taken in this study is the methodology proposed by Andrés Calle, Cascón, and González-Arteaga in [8] that it was especially designed for such aim. Data for this study were collected from Spanish Health Barometer survey and they were searched from 1995 until 2018. The sample was representative with respect to gender, age and regions, including interviews of 161,163 randomly chosen Spanish people. The experimental work presented here provides one of the first investigations into how Spaniards’ preferences about Healthcare system change over time.

This work is composed of five sections. The first section of this paper examines the structure and the basic characteristics of the public healthcare system in Spain. Section 3 gives a brief overview of the starting hypothesis as well as the methodology used in the paper. Section 4 analyses the results of interviews and focus on measuring stability of preferences focusing on the three key scenarios analyzed. Section 5 presents the findings and the conclusions of the research. Appendix A contains tables and figures.

## 2. The Public Healthcare System in Spain

**Figure 1:**
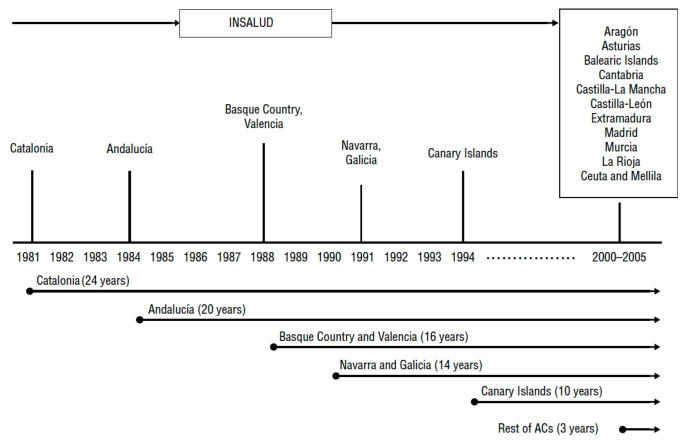
Chronology of devolution of health competences to autonomous regions in Spain. * Source: Ministry of Health and Consumer Affairs [9].

Nowadays, the Spanish healthcare system is a universal coverage system (including irregular immigrants) that is almost entirely supported by the public sector. The political organization of Spain is composed of the central state and 17 highly decentralized regions (Autonomous Communities). Health competences were completely transferred to the regional authorities at the end of 2002. This transition process started in 1981 from a centralized model of health services to 17 regional health services (see Figure 1). The financing of the autonomous regions is independent of the central state in expenditure and fundraising. The central authority only supports certain strategic areas on Healthcare like pharmaceuticals’ legislation and also it looks out for the equitable functioning of health services across the country.

There have been three main health reforms. The first took place in the 1980s, and it was predominantly aimed at the extension of the coverage and access to healthcare services, i.e., a universal national health system, due to the fact that the preceding system was a limited social security system. The second one was developed in the 1990s and it was focused on keeping costs under control and managing innovation. The last reform was implemented in the 2000s and its main focal point was the coordination among regions after healthcare services devolution.

In 2017, total healthcare expenditure in Spain (all the data is made available on https://ec.europa.eu/eurostat/statistics-explained/index.php?title=Healthcare_expenditure_statistics) was 2221 € per capita and 8.87% of its GDP (103 billion €), which is below the European average (2887 € and 9.9% GDP). As aforementioned, 71% of the health expenditure depends on the public sector (funded mostly from taxation). From 2008 to 2014 public spending on healthcare was reduced due to the economic crisis. Particularly, the Spanish Healthcare system was significantly affected by cutting staff and salary reduction of the healthcare personnel, and by reducing the scope of the public health insurance. Furthermore and due to the decentralization of the healthcare system, there are significant differences among regions, not only in health expenditure but also in practitioners, staff and health workers, healthcare services and hospital waiting times.

On another note, life expectancy in 2017 was 83.4 years in Spain, the highest in Europe. This item has been increased over the years. Since 2000 it has risen by more than four years. This fact has brought about an increasing long-term care expenditure in Spain (see [3]).

Considering all of these aspects, one may suppose that Spanish citizens could change their opinions and preferences on public healthcare institution over the years. The section that follows moves on to consider the stability of such preferences from 1995 to 2018.

## 3. Starting Points, Methodology, and Data Sources

This paper researches the stability of Spaniards’ preferences about their public healthcare system from 1995 to 2018. For such a purpose it is necessary to establish some previous For such a purpose it is necessary to establish some previous assumptions, which is the focus of this section.

First of all, the way to define individuals preferences as well as the methodology used to measure preferences stability are presented. Then, data sources and sample characteristic used in this research are expounded.

### 3.1. About Individuals Preferences

People’s preferences are often characterized by their ideas, principles, knowledge, and so on. In each stage of time, which causes difficulties when it comes to measuring their stability. The Arrovian position assumes that each individual constructs a preference binary relation (usually a weak order) on the set of alternatives by using some unspecified internal process [10]. Following Arrovian assumptions, this research considers that individuals express their preferences on a finite set of alternatives and it is assumed that citizens rank such alternatives by means of complete pre-orders (reflexive and transitive binary relation) in order to be faithful to reality.

Let N={1,…,n} a society of individuals and $X=\{x_{1},\ldots ,x_{k}\}$ a finite set of alternatives, ∣X∣≥2. Let $T=\{t_{0},\ldots ,t_{T}\}$ be an ordered time sequence, namely, a *temporal set*.

W(X) denotes the set of all complete pre-orders on X. Let $R_{i}\in W\left(X\right)$ be a *temporal preference* on the set of alternatives X at the moment of time $t_{i}\in T$. Therefore, the notation $x_{k}R_{i}x_{j}$ means that the alternative $x_{k}$ is at least as good as $x_{j}$ at the moment of time $t_{i}$.

Let $P=\left(R_{0},R_{1},\ldots ,R_{T}\right)\in W\left(X\right)\times \ldots \times W\left(X\right)=W{\left(X\right)}^{T+1}$ be a *temporal preference profile* on the set of alternatives X, where $R_{i}\in P$ represents the temporal preference on X at the moment of time $t_{i},i\in \left\{0,1,\ldots ,T\right\}$.

### 3.2. Methodology

As far as we know, there are few studies that have investigated how to measure the stability of temporal preferences (see in [11,12,13]). In this contribution, the methodology proposed by de Andrés Calle, Cascón and González-Arteaga in [8] is used for measuring changes on preferences over time. This approach is particularly useful in measuring how much preferences change over time when preferences are considered as complete pre-orders. The approach includes two particular measures: the *local* and the *global preference stability measure*. Both measures verify several mathematical properties, such as *full preference stability* (the measure of stability must be maximum if preferences over time are equal) and *preference stability neutrality* (the measure of stability must not change if the alternatives are permuted), that ensure their robustness. Furthermore, the model includes a parameter, the λ-*parameter*, that depicts the *memory loss effect* of people on preferences over time. In this regard, when λ>0, individuals express their preferences on the set of alternatives but taking into account more intensively the most recent temporal decisions; when λ=0, individuals express their preferences on the set of alternatives but taking equally into account the previous temporal decisions. These definitions are formulated hereunder.

**Definition** **1** [8]**.** *Let $P=\left(R_{0},\ldots ,R_{T}\right)\in W{\left(X\right)}^{T+1}$ be a temporal decision profile. The* local preference stability measure *between the temporal preferences at the moments of time $t_{i-1}$ and $t_{i}$ is a mapping $\theta_{i}:W\left(X\right)⟶\left[0,1\right]$ given by*

$$\theta_{i}\left(P\right)=\theta_{\left[i-1,i\right]}\left(P\right)=1-\frac{\|c_{R_{i-1}}-c_{R_{i}}{\|}_{1}}{r}$$

*where $c_{R_{i-1}}$ and $c_{R_{i}}$ are the canonical codified vectors associated to the preferences $R_{i-1}$ and $R_{i}$, respectively; ${\left\|\;\cdot \;\right\|}_{1}$ denotes the l${}_{1}$-norm, then*

*$\|c_{R_{i-1}}-c_{R_{i}}{\|}_{1}=\sum_{h=1}^{k}|c_{h}^{R_{i-1}}-c_{h}^{R_{i}}|$; and finally, $r=\max_{c,c^{\prime }\in F}{\left\|c-c^{\prime }\right\|}_{1}$. Therefore,*

$$\theta_{i}\left(P\right)=\theta_{\left[i-1,i\right]}\;\;\left(P\right)=1-\frac{\sum_{h=1}^{k}|c_{h}^{R_{i-1}}-c_{h}^{R_{i}}|}{\max_{c,c^{\prime }\in F}{\left\|c-c^{\prime }\right\|}_{1}}$$

The local preference stability measure estimates the stability between two consecutive temporal preferences as one minus the proportion of the number of preferences changes over the maximum that could happen between such two consecutive moments of time. Taking into account such values, the global preference stability measure is the λ-*weighted mean* of the local preference stability measures over time.

**Definition** **2** [8]**.** *Let $P\in W{\left(X\right)}^{T+1}$ a decision temporal profile. The* global preference stability measure *for the temporal preference profile $P\in W{\left(X\right)}^{T+1}$ is the mapping $\Theta :W{\left(X\right)}^{T+1}\times R^{+}⟶\left[0,1\right]$ given by*

$$\Theta \left(P,\lambda \right)=\sum_{i=1}^{T}w_{i,T}\left(\lambda \right)\cdot \theta_{i}\left(P\right)$$

*where*

$$w_{i,T}\left(\lambda \right)=A_{T}\left(\lambda \right)\cdot e^{-\lambda (T-i)}\;and\;A_{T}\left(\lambda \right)=\left\{\begin{array}{cc} \frac{1-e^{-\lambda }}{1-e^{-\lambda T}} & if\;\lambda >0,\\ \frac{1}{T} & if\;\lambda =0. \end{array}\right.$$

In this contribution to compute the global preference stability measure and to analyse the effect of the λ-parameter, i.e., the *memory loss effect* of the Spanish society, different values for λ-parameter are considered, in particular, λ={0,0.1,0.25,0.5,0.75}.

### 3.3. Data Sources and Sample Characteristics

Data regarding Spaniards’ opinions and their perceptions on public health system were obtained by means of the survey *Health Care Barometer* (all data are available through the website http://www.cis.es/cis/opencm/EN/2_bancodatos/estudios/listaTematico.jsp?tema=112&todos=no) compiled by the *Spanish Center of Sociological Research* (CIS), first used in 1995. The CIS carries out this survey on a quarterly basis (February, June and November) and it includes interviews from around 7800 randomly chosen Spanish people per year. The interviews are face-to-face (household) and they incorporate 75 questions: 43 of them are fixed (citizens’ opinions on their healthcare experience are included here), 15–20 are on an ad hoc basis, and 15–18 are socio-demographic questions. Focusing on questions about healthcare experience, aspects like access to public healthcare system, patient safety, satisfaction, opinions on health system, an so on are covered.

Particularly, this contribution includes interviews for 161,163 randomly chosen people from all over the country and for each year from 1995 to 2018. More specifically, Table 1 shows the different samples sizes for each year.

The CIS carries out a multi-stage stratified random sampling to collect the Healthcare Barometer data. Specifically, the basic sampling units (regions and municipalities) are selected in a proportional random way and the secondary sampling units (individuals) are selected by random routes and gender-age quotas (these specific aspects are available through the website http://www.cis.es/cis/export/sites/default/-Archivos/Marginales/3220_3239/3227/FT3227.pdf). With respect to the different regions (CCAA), the Spanish Center of Sociological Research uses sample sizes proportional to the length of time but are not proportional to the population. The proportions (note the elimination of Ceuta and Melilla regions due to the fact that they was recently joined the sample) used are shown in Table 2.

**Figure 2:**
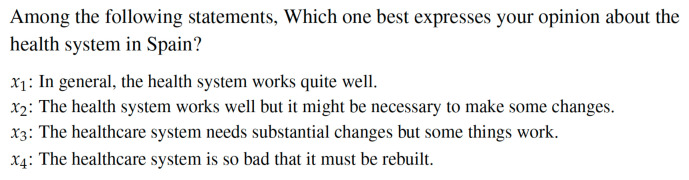
Question 1 under study.

The Spanish society by means of the Health Barometer survey reports diverse questions related to its satisfaction and opinion on public health services as commented on. This study focuses into two particular questions from such a questionnaire. The first one alludes to a general opinion of the health system. Spaniards must select among four alternatives establishing then their temporal preferences on the set of alternatives for each year (see Figure 2). By means of Question 1, Spaniards choose which alternative is the best for them and explains better the Spanish healthcare system. According to their answers, the social preferences on the four alternatives are established for each year from 1995 to 2018.

**Figure 3:**
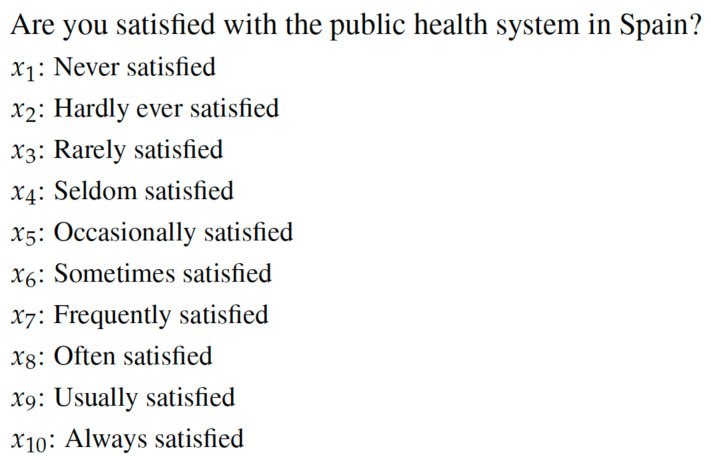
Question 2 under study.

The second question under study is related to the level of satisfaction with public health system and it was introduced in the Health Care Barometer in 2001. In this case, citizens must choose among ten alternatives from “never satisfied” to “always satisfied”. By this question Spanish people choose which alternative is the best for them and better explains their satisfaction with the public healthcare system (see Figure 3). According to their answers, the social preferences on the ten alternatives are established for each year. For this particular case, the years under analysis are from 2001 to 2018.

Bringing these questions, and the characteristics of the Spanish healthcare system, into focus, they can be addressed best under the following three headings.

- First, it seems interesting to study how much Spaniards’ preferences are stable but considering the entire society.
- Second, and due to the decentralization of the healthcare system, it could be engrossing to measure the stability of the preferences bearing in mind the different Spanish regions.
- Finally and because of citizens’ perceptions about the healthcare system could depend on the age, it could be therefore appropriate to analyze the preferences considering the population depending on its age. In this regard, three age groups have been considered: younger than 34 years old, between 35–54 years old and older than 55 years old.

## 4. Results

### 4.1. Analysis of Question 1: Have Spaniards’ Preferences on the Health System Changed?

#### 4.1.1. Analysis of the Society

Through Question 1 (see Figure 2) Spanish society establishes its temporal preferences about the public healthcare system from 1995 to 2018. These preferences are shown in Table A1.

**Figure A1:**
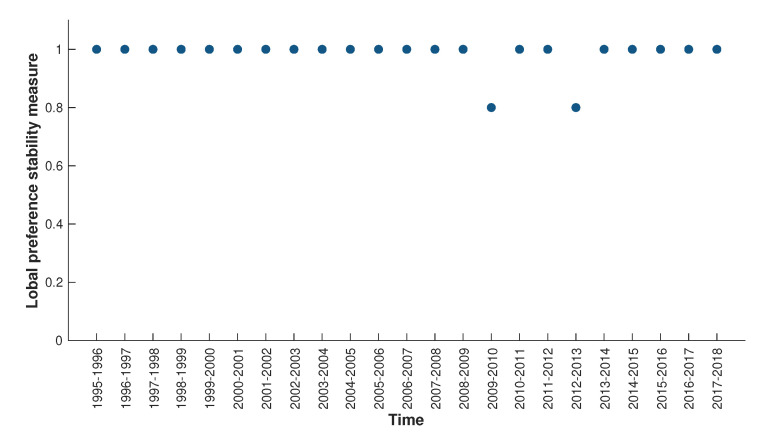
Graphic illustration of the local decision stability measures obtained for the entire society for Question 1.

Once the preferences have been established, the local and the global preference stability measures can be computed by Definitions 2 and 3 from [8], respectively. Figure A1 provides an overview of the local decision stability measures obtained for the entire society.

**Figure A2:**
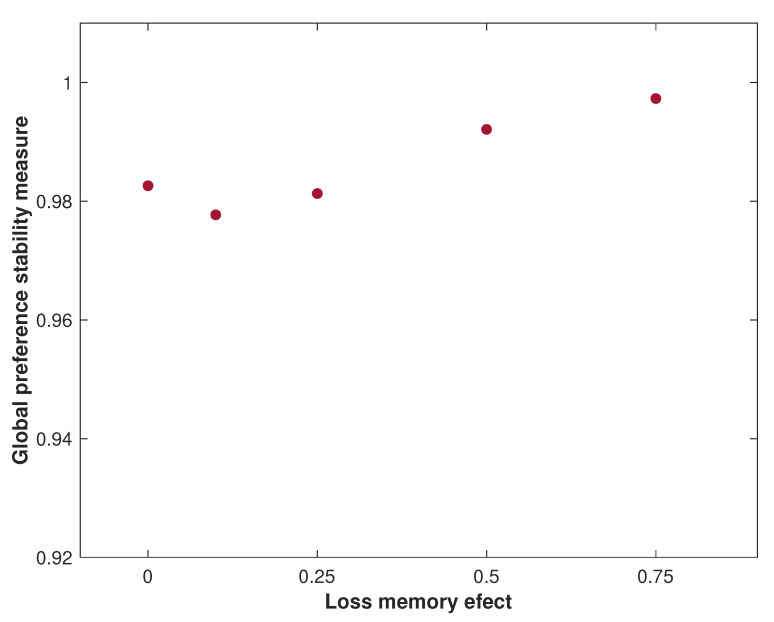
Graphic illustration of the global preferences stability measures for the entire society from different values of λ for Question 1.

As can be seen from data in Table A2, and as might be expected, general Spaniards’ preferences on the public health care system are very stable. Most of the global preference stability measures for several values of λ are close to 1, i.e., the results show a high level of stability of the Spanish society’s opinion on the public healthcare system, considering that “the health system works well but it might be necessary to make some changes”. Figure A2 provides an overview of the global preference stability measures obtained for the entire population.

#### 4.1.2. Analysis by Regions

**Figure A3:**
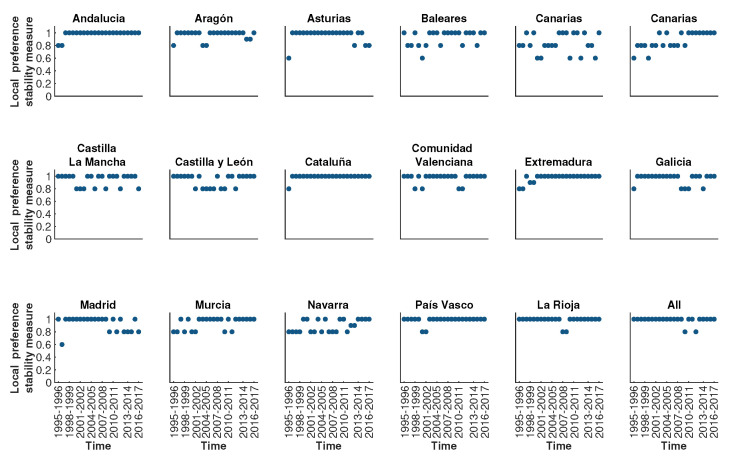
Graphic illustration of the local decision stability measures for Question 1 obtained for each region.

**Figure A4:**
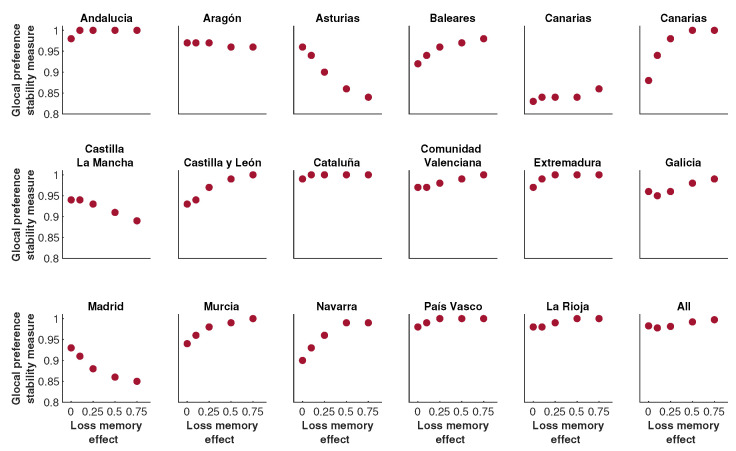
Graphic illustration of the global decision stability measures for Question 1 obtained for each region.

The temporal preferences for Question 1 to the different regions have been gathered in a similar way as in previous analysis but they have been omitted due to the large size of the tables (all of them are available on request). The local stability preferences measures obtained from the analysis are presented in the Figure A3. The global stability measures are summarized in Table A3 and in Figure A4. From plots Figure A3 and Figure A4, it is clear that there are differences among the values of the stability of the regional groups. It could be noted that some regions, like Asturias and Madrid, present high local stability but not global stability; in both, stability decreases as the memory loss effect increases. There are also regions that present high partial and global stability, such as Andalucía, Aragón, Cataluña, País Vasco and La Rioja. Moreover, some regions present more instability, in local and global measures, like Canarias and Madrid.

#### 4.1.3. Analysis by Age-Group

**Figure A5:**
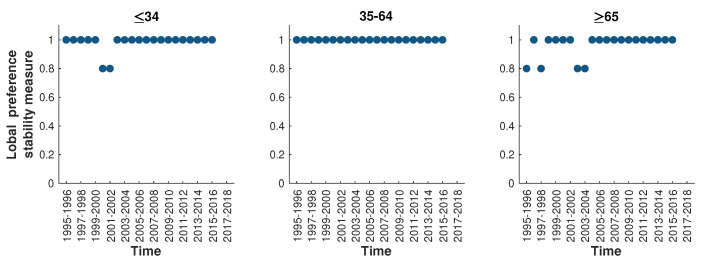
Graphic illustration of the local decision stability measures obtained for each age group for Question 1.

Table A4 presents the temporal preferences collected depending on the age ranges. No significant differences were found among age groups’ preferences as Table A4 shows. Figure A5 provides an overview of the local decision stability measures obtained for each age group. What can be clearly emphasized in this figure is the local full preference stability for the age group between 35–54 years old.

**Figure A6:**
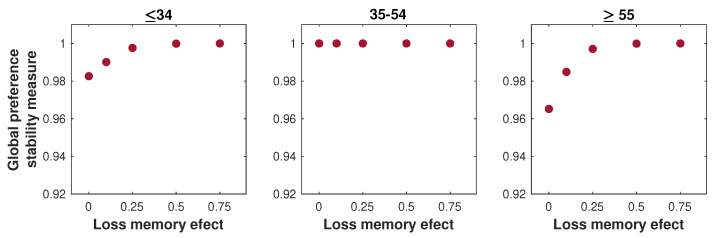
Graphic illustration of the global preferences stability measures from different values of λ and different age ranges for Question 1.

The global preference stability measures obtained from the analysis of such preferences are summarized in Table A5. Figure A6 provide an overview of the global preference stability measures obtained for each age group. As might be expected, Table A5 reveals that there has been a marked full preference stability in the age group 35–54. Additionally, it is apparent that there is not a substantial difference between the age groups younger than 34 and older than 55.

As far as age groups are concerned, the close proximity of the value of their stability measures is striking although age groups’ preferences do not match. This fact makes it worth computing the agreement, or the lack of it, among age groups’ preferences. To this end, it is necessary to determine a representative preference for each age group before measuring how much consensus or dissensus there is among them. All these computations require proper methodologies. Given that in this contribution the preferences are expressed like complete pre-orders and the model from [8] has been assumed, it is coherent to adopt the methodology presented in [14,15]. These works approach the consensus or dissensus measurement for a collection of complete pre-orders based on the Mahalanobis distance and they also present the development of a social consensus solution as an appropriate representative from an optimization problem.

Taking into account these methodologies and focusing on Table A4, it is possible to compute the representative preference (the representative preference was calculated by using the identity matrix as Σ) for each age group by means of the approach presented in [15]. Therefore, the representative preference for younger than 34 is $x_{2}≻x_{3}≻x_{1}≻x_{4}$, between 35 and 54 is $x_{2}≻x_{3}≻x_{1}≻x_{4}$, and for older than 55 is $x_{2}≻x_{1}≻x_{3}≻x_{4}$. Note that the representative preference from citizens older than 55 years of age is different from others and that the representative preference for the other two age group coincides. In detail, this result means that overall elder people give precedence to alternative $x_{1}$: “*In general, the health system works quite well”* over alternative $x_{3}$: “*The healthcare system needs substantial changes, but some things work*”, in contrast to the rest of age groups. This output was expected after watching preferences in Table A4.

For the purpose of completing the analysis, the degree of consensus among the representative preferences of the age groups has been computed by the methodology proposed in [14]. Therefore, the dissensus measure among the three age group representatives’ preferences is 1.33, that is a value near zero which means that the dissensus among them is low (the dissensus measure proposed in [14] is defined on the interval [0,∞)).

### 4.2. Analysis of Question 2: Have the Spaniards’ Level of Satisfaction with the Public Health System Changed?

#### 4.2.1. Analysis of the Society

**Figure A7:**
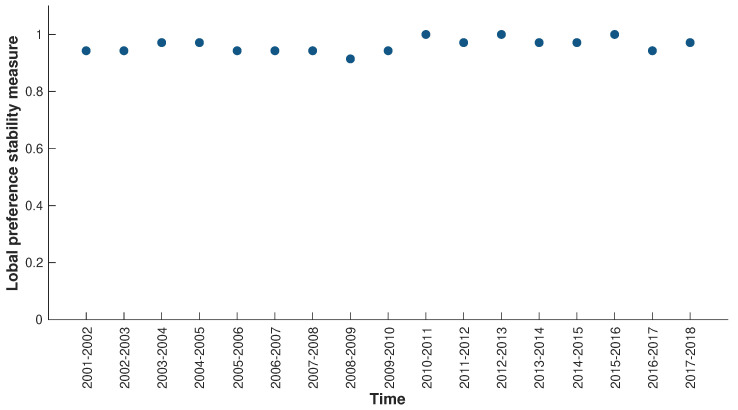
Graphic illustration of the local decision stability measures obtained for the entire society for Question 2.

Table A6 gathers citizens’ temporal preferences about their satisfaction with public healthcare system for each year. Figure A7 presents the results obtained from computing the local decision stability measures for the entire society. From this chart, it can be seen that by far the greatest local stability is for the year 2011 although the local stability values are fairly similar to each other.

What stands out in Table A7 is the high level of stability of the society’s opinion on their satisfaction with public healthcare system. Interestingly, most of the years alternative $x_{7}$, “Frequently satisfied”, is ordered in the first place notwithstanding the number of alternatives.

**Figure A8:**
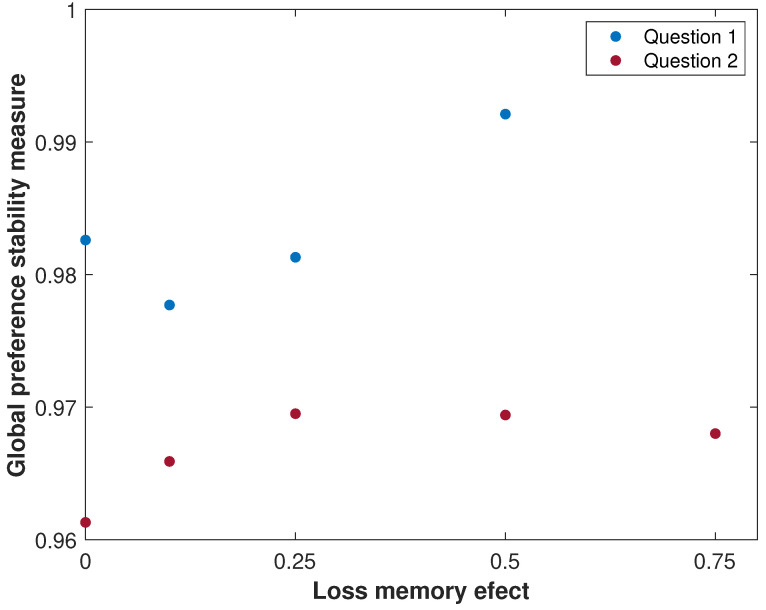
Graphic illustration of the global preferences stability measures for Spaniards from different values of λ for Questions 1 and 2.

The value of the global stability measure obtained in Question 2 is smaller than in Question 1 for each value of λ. The differences between the values of the global stability measures from Question 1 and Question 2 are highlighted in Figure A8.

Moving on now to consider the analysis of the questions by regions.

#### 4.2.2. Analysis by Regions

The temporal preferences for Question 2 of the different regions have been gathered in a similar way as in the previous analysis although because of the large size of the tables, they have been omitted (all of them are available on request).

**Figure A9:**
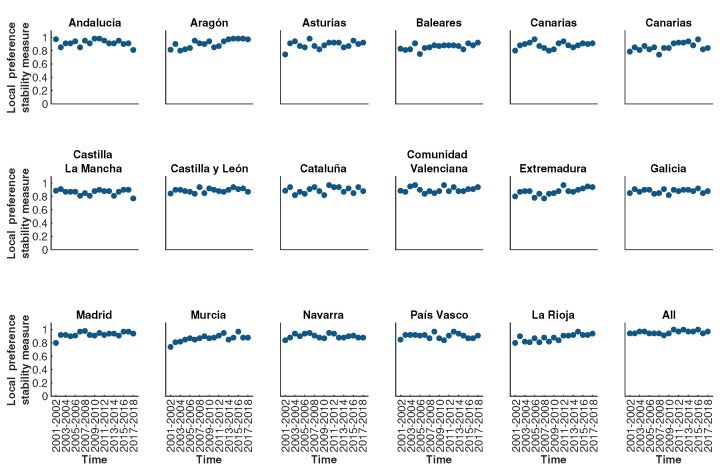
Graphic illustration of the local decision stability measures for Question 2 obtained for each region.

**Figure A10:**
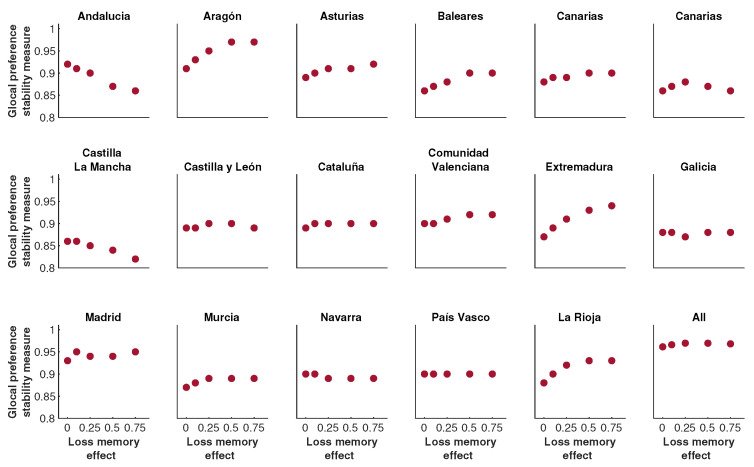
Graphic illustration of the global decision stability measures for Question 2 obtained for each region.

The results of the local stability measures for each region are shown in Figure A9. There are several differences among regions preferences. There are remarkable high values of this measure for those regions of which were firstly transferred health competences like Andalucía, Cataluña and País Vasco (see Figure 1). The most local instability appears in Baleares, Canarias and also Cantabria (Baleares and Cantabria achieve health competences in the 2000–2005 period). In the same vein the local stability, no difference greater than global stability were observed as can be shown in Figure A10.

From Table A8, it is evident that there are differences among regions. It could be noted that some regions, like Navarra and País Vasco, present high stability regardless of the memory loss effect, but other communities change their stability based on the memory loss effect.

#### 4.2.3. Analysis by Age-Group

Turning now to the analysis by age group, age groups’ preferences from 2001 to 2018 show that citizens’ preferences are focused on alternative $x_{7}$, in a similar way as in the analysis of the society although some differences can been observed. Due to the large size of the tables, as in the previous question the temporal preferences for Question 2 have been omitted (all of them are available on request).

**Figure A11:**
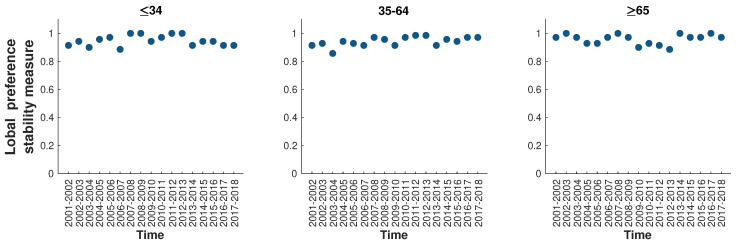
Graphic illustration of the local decision stability measures obtained for each age group and Question 2.

**Figure A12:**
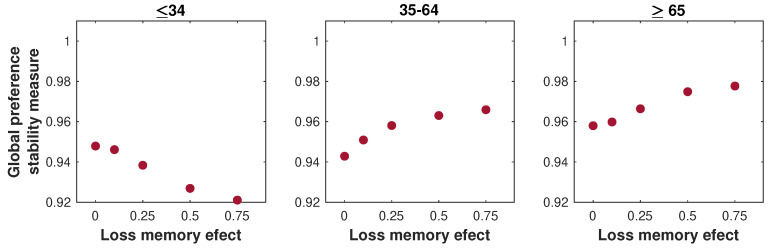
Graphic illustration of the global preferences stability measures from different values of λ and different age ranges for Question 2.

Taking into account these preferences, Figure A11 provides an overview of the local decision stability measures obtained for each age group. The global stability measures obtained from the analysis of these preferences are summarized in Table A9 and Figure A12. What is interesting about the values in Table A9 is that the global stability measure decreases as the memory loss effect increases in younger citizens while the global stability measure of the other groups performs in the opposite way. Additionally, it can be observed that all age groups present high stability, being the group over 54 the one with highest stability measure.

As was pointed out in the case of Question 1, high stability does not mean equivalence of age groups preferences. For this reason, as well as in Question 1, it could interesting to compute the representative preference of each group. Table 3 contains the representative preferences (the computation is made using the methodology in [15] with Σ the identity) for each age group that which have been determined by using the methodology presented in [15].

To complete the analysis the dissensus measure for the three age group has been computed, which is 16. This result is greater than the corresponding for Question 1 and the dissensus measure between the groups ≤34 and 35–54 is lower than the dissensus measure between the groups ≤34 and ≥55. This implies that the preferences of the age groups ≤34 and 35–54 are closer than the preferences of the age groups ≤34 and ≥55, and also than 35–54 and ≥55.

## 5. Discussion

In this study, the preferences of Spaniards on their healthcare system are considered in a new perspective, the evaluation of their stability over the time. The main findings are that the preference stability measurements show very high values over the years for the studied issues irrespective of the age group or the region.

This investigation makes use of the available information drawn from the Spanish Health Care Barometer. More specifically, two questions are focused on in this study: the general opinion of the public healthcare system and the level of satisfaction with it. These questions ask citizens for a global evaluation of the healthcare system according to their perceptions.

In accordance with the characteristic of the Spanish healthcare system, the research was carried out by means of three rubrics: the entire society, the different Spanish regions and paying attention to the age. In all these cases, preferences on the public healthcare system are very stable over the years for the two studied questions. This is a notable outcome.

Although a high preferences stability measure is found, it does not mean that the preferences coincide in each age group and region. Likewise, the derived representative of preference relies on age groups for the two studied questions. These findings suggest that preferences on the healthcare system depend on the age and the use of it in each life stage.

Regarding the analysis by regions, it is observed that in several years instability has grown, which could be consequence of the fall in investments due to the economic crisis and also the competences decentralization process.

In connection with the methodology, it is interesting to discuss the role of the parameter λ, i.e., the memory loss effect. It can be viewed as a shortcoming of our proposal. Nevertheless, it is not a remarkable point in the obtained results in this study.

In future studies, it may be necessary to take into account other factors in order to gain further insight into the stability of the preferences. Among those factors is the education level, since the differences in the education level could affect citizens’ perceptions about the healthcare system. Therefore, further research could be undertaken to investigate how this and other factors affect citizens’ preferences.

## 6. Conclusions

As is well known, the health system is a key feature for countries, and due to this fact there is increasing interest in knowing its performance and how citizens appreciate it. Therefore, in recent decades different methodologies and surveys have emerged to know the opinions and preferences of the Spanish people, notable among these is the Spanish Health Care Barometer survey. This survey was conducted to obtain representative samples of the Spanish population and it is used in this study.

The results show that for the entire society Spaniards’ preferences on the public healthcare system have been very stable over time. It can be seen that by far the greatest local stability is for the year 2011 although the local stability values are fairly similar to each other. In general terms, the value of the global stability measure obtained for the question on the level of satisfaction is smaller than for the question of the general opinion.

The results are analogous if the analysis is made by region, especially for the question of the general opinion. However, there are some regional differences among the stability measures for the level of satisfaction and there are remarkable high values of this measure for those regions of which were firstly transferred health competences.

It is also obtained high stability values in each age group for the two studied issues. In relation to the general opinion question, there are not a substantial differences between the stability measures in the younger and older ones.

In conclusion, the preferences of Spaniards on their healthcare system show high stability regarding the level of satisfaction and general opinion irrespective of the age group or the region.

## Figures and Tables

**Table 1:** Sample size for each year.

Year	Sample Size	Year	Sample Size	Year	Sample Size
1995	6759	2003	6785	2011	7757
1996	2263	2004	6759	2012	7729
1997	4524	2005	6728	2013	7750
1998	6778	2006	6756	2014	7721
1999	6786	2007	6745	2015	7746
2000	6773	2008	7125	2016	7752
2001	2257	2009	7752	2017	7736
2002	6746	2010	7750	2018	7686

**Table 2:** Proportions used by the Spanish Center of Sociological Research to obtain regional sample sizes.

Regions	Proportion	Regions	Proportion
Andalucia	10.2%	Comunidad Valenciana	7.4%
Aragón	4.4%	Extremadura	4.2%
Asturias	4.2 %	Galicia	5.7%
Baleares	4.2 %	Madrid	8.5%
Canarias	4.9%	Murcia	4.1%
Cantabria	3.8%	Navarra	3.7%
Castilla La Mancha	5%	País Vasco	5%
Castilla y León	5.3%	La Rioja	3.4%
Cataluña	9.6%		

**Table 3:** Representative preferences attending to different age ranges for Question 2.

Age-Group	Preferences
≤34	$X_{7}≻X_{6}≻X_{5}≻X_{8}≻X_{4}≻X_{9}≻X_{3}≻X_{1}≻X_{10}≻X_{2}$
35–54	$X_{7}≻X_{6}≻X_{8}≻X_{5}≻X_{4}≻X_{9}≻X_{3}≻X_{10}≻X_{1}≻X_{2}$
≥55	$X_{8}≻X_{7}≻X_{6}≻X_{5}≻X_{9}≻X_{10}≻X_{4}≻X_{3}≻X_{1}≻X_{2}$

## Appendix A

**Table A1:** Temporal preferences established by Healthcare Spanish Barometer for Question 1.

Year	Spaniards’ Preferences	Year	Spaniards’ Preferences
1995	$x_{2}≻x_{3}≻x_{1}≻x_{4}$	2007	$x_{2}≻x_{3}≻x_{1}≻x_{4}$
1996	$x_{2}≻x_{3}≻x_{1}≻x_{4}$	2008	$x_{2}≻x_{3}≻x_{1}≻x_{4}$
1997	$x_{2}≻x_{3}≻x_{1}≻x_{4}$	2009	$x_{2}≻x_{3}≻x_{1}≻x_{4}$
1998	$x_{2}≻x_{3}≻x_{1}≻x_{4}$	2010	$x_{2}≻x_{1}≻x_{3}≻x_{4}$
1999	$x_{2}≻x_{3}≻x_{1}≻x_{4}$	2011	$x_{2}≻x_{1}≻x_{3}≻x_{4}$
2000	$x_{2}≻x_{3}≻x_{1}≻x_{4}$	2012	$x_{2}≻x_{1}≻x_{3}≻x_{4}$
2001	$x_{2}≻x_{3}≻x_{1}≻x_{4}$	2013	$x_{2}≻x_{3}≻x_{1}≻x_{4}$
2002	$x_{2}≻x_{3}≻x_{1}≻x_{4}$	2014	$x_{2}≻x_{3}≻x_{1}≻x_{4}$
2003	$x_{2}≻x_{3}≻x_{1}≻x_{4}$	2015	$x_{2}≻x_{3}≻x_{1}≻x_{4}$
2004	$x_{2}≻x_{3}≻x_{1}≻x_{4}$	2016	$x_{2}≻x_{3}≻x_{1}≻x_{4}$
2005	$x_{2}≻x_{3}≻x_{1}≻x_{4}$	2017	$x_{2}≻x_{3}≻x_{1}≻x_{4}$
2006	$x_{2}≻x_{3}≻x_{1}≻x_{4}$	2018	$x_{2}≻x_{3}≻x_{1}≻x_{4}$

**Table A2:** Global stability measures for Spaniards’ preferences attending to several values of λ for Question 1.

	λ				
	**0**	**0.1**	**0.25**	**0.5**	**0.75**
Global preference stability	0.9826087	0.9776651	0.9812783	0.9920990	0.9972567

**Table A3:** Global stability measures for Spaniards’ preferences attending to different regions and several values of λ for Question 1.

	λ				
**Regions**	**0**	**0.1**	**0.25**	**0.5**	**0.75**
Andalucia	0.9826087	0.9950658	0.9995857	00.9999965	1.0000000
Aragón	0.9652174	0.9684452	0.9660163	0.9614682	0.9632942
Asturias	0.9565217	0.9408391	0.9043652	0.8629218	0.8393721
Baleares	0.9217391	0.9437514	0.9578652	0.9668629	0.9752670
Canarias	0.8347826	0.8376190	0.8355414	0.8417044	0.8601247
Cantabria	0.8782609	0.9356513	0.9802530	0.9973639	0.9996352
Castilla la Mancha	0.9391304	0.9381899	0.9335265	0.9136905	0.8918526
Castilla y León	0.9304348	0.9404001	0.9676798	0.9904916	0.9970828
Cataluña	0.9913043	0.9976561	0.9998186	0.9999987	1.0000000
Comunidad Valenciana	0.9652174	0.9708581	0.9813679	0.9936837	0.9982736
Extremadura	0.9739130	0.9917355	0.9991472	0.9999887	0.9999999
Galicia	0.9565217	0.9533758	0.9604581	0.9777476	0.9879387
Madrid	0.9304348	0.9053828	0.8750345	0.8587822	0.8531169
Murcia	0.9391304	0.9626528	0.9818467	0.9945889	0.9985651
Navarra	0.8956522	0.9257459	0.9597434	0.9855999	0.9947421
País Vasco	0.9826087	0.9918649	0.9985541	0.9999576	0.9999990
La Rioja	0.9826087	0.9836178	0.9916792	0.9985955	0.9998181

**Table A4:** Temporal preferences established by Healthcare Spanish Barometer attending to different age ranges for Question 1.

Age-Group			
**Year**	≤34	**35–54**	≥55
1995	$x_{2}≻x_{3}≻x_{1}≻x_{4}$	$x_{2}≻x_{3}≻x_{1}≻x_{4}$	$x_{2}≻x_{1}≻x_{3}≻x_{4}$
1996	$x_{2}≻x_{3}≻x_{1}≻x_{4}$	$x_{2}≻x_{3}≻x_{1}≻x_{4}$	$x_{1}≻x_{2}≻x_{3}≻x_{4}$
1997	$x_{2}≻x_{3}≻x_{1}≻x_{4}$	$x_{2}≻x_{3}≻x_{1}≻x_{4}$	$x_{1}∼x_{2}≻x_{3}≻x_{4}$
1998	$x_{2}≻x_{3}≻x_{1}≻x_{4}$	$x_{2}≻x_{3}≻x_{1}≻x_{4}$	$x_{2}≻x_{1}≻x_{3}≻x_{4}$
1999	$x_{2}≻x_{3}≻x_{1}≻x_{4}$	$x_{2}≻x_{3}≻x_{1}≻x_{4}$	$x_{2}≻x_{1}≻x_{3}≻x_{4}$
2000	$x_{2}≻x_{3}≻x_{1}≻x_{4}$	$x_{2}≻x_{3}≻x_{1}≻x_{4}$	$x_{2}≻x_{1}≻x_{3}≻x_{4}$
2001	$x_{2}≻x_{3}≻x_{1}≻x_{4}$	$x_{2}≻x_{3}≻x_{1}≻x_{4}$	$x_{2}≻x_{1}≻x_{3}≻x_{4}$
2002	$x_{2}≻x_{3}≻x_{1}≻x_{4}$	$x_{2}≻x_{3}≻x_{1}≻x_{4}$	$x_{2}≻x_{1}≻x_{3}≻x_{4}$
2003	$x_{2}≻x_{1}≻x_{3}≻x_{4}$	$x_{2}≻x_{3}≻x_{1}≻x_{4}$	$x_{2}≻x_{3}≻x_{1}≻x_{4}$
2004	$x_{2}≻x_{3}≻x_{1}≻x_{4}$	$x_{2}≻x_{3}≻x_{1}≻x_{4}$	$x_{2}≻x_{1}≻x_{3}≻x_{4}$
2005	$x_{2}≻x_{3}≻x_{1}≻x_{4}$	$x_{2}≻x_{3}≻x_{1}≻x_{4}$	$x_{2}≻x_{1}≻x_{3}≻x_{4}$
2006	$x_{2}≻x_{3}≻x_{1}≻x_{4}$	$x_{2}≻x_{3}≻x_{1}≻x_{4}$	$x_{2}≻x_{1}≻x_{3}≻x_{4}$
2007	$x_{2}≻x_{3}≻x_{1}≻x_{4}$	$x_{2}≻x_{3}≻x_{1}≻x_{4}$	$x_{2}≻x_{1}≻x_{3}≻x_{4}$
2008	$x_{2}≻x_{3}≻x_{1}≻x_{4}$	$x_{2}≻x_{3}≻x_{1}≻x_{4}$	$x_{2}≻x_{1}≻x_{3}≻x_{4}$
2009	$x_{2}≻x_{3}≻x_{1}≻x_{4}$	$x_{2}≻x_{3}≻x_{1}≻x_{4}$	$x_{2}≻x_{1}≻x_{3}≻x_{4}$
2010	$x_{2}≻x_{3}≻x_{1}≻x_{4}$	$x_{2}≻x_{3}≻x_{1}≻x_{4}$	$x_{2}≻x_{1}≻x_{3}≻x_{4}$
2011	$x_{2}≻x_{3}≻x_{1}≻x_{4}$	$x_{2}≻x_{3}≻x_{1}≻x_{4}$	$x_{2}≻x_{1}≻x_{3}≻x_{4}$
2012	$x_{2}≻x_{3}≻x_{1}≻x_{4}$	$x_{2}≻x_{3}≻x_{1}≻x_{4}$	$x_{2}≻x_{1}≻x_{3}≻x_{4}$
2013	$x_{2}≻x_{3}≻x_{1}≻x_{4}$	$x_{2}≻x_{3}≻x_{1}≻x_{4}$	$x_{2}≻x_{1}≻x_{3}≻x_{4}$
2014	$x_{2}≻x_{3}≻x_{1}≻x_{4}$	$x_{2}≻x_{3}≻x_{1}≻x_{4}$	$x_{2}≻x_{1}≻x_{3}≻x_{4}$
2015	$x_{2}≻x_{3}≻x_{1}≻x_{4}$	$x_{2}≻x_{3}≻x_{1}≻x_{4}$	$x_{2}≻x_{1}≻x_{3}≻x_{4}$
2016	$x_{2}≻x_{3}≻x_{1}≻x_{4}$	$x_{2}≻x_{3}≻x_{1}≻x_{4}$	$x_{2}≻x_{1}≻x_{3}≻x_{4}$
2017	$x_{2}≻x_{3}≻x_{1}≻x_{4}$	$x_{2}≻x_{3}≻x_{1}≻x_{4}$	$x_{2}≻x_{1}≻x_{3}≻x_{4}$
2018	$x_{2}≻x_{3}≻x_{1}≻x_{4}$	$x_{2}≻x_{3}≻x_{1}≻x_{4}$	$x_{2}≻x_{1}≻x_{3}≻x_{4}$

**Table A5:** Global stability measures for Spaniards’ preferences attending to different age ranges and several values of λ for Question 1.

	λ				
**Age-Group**	**0**	**0.1**	**0.25**	**0.5**	**0.75**
≤34	0.9826087	0.9900637	0.9976161	0.9998847	0.9999957
35–54	1	1	1	1	1
≥55	0.9652174	0.9848571	0.9971357	0.9998798	0.9999957

**Table A6:** Temporal preferences established by Healthcare Spanish Barometer from Question 2.

Year	Spaniards’ Preferences
2001	$x_{7}≻x_{5}≻x_{6}≻x_{8}≻x_{4}≻x_{9}≻x_{3}≻x_{10}≻x_{1}≻x_{2}$
2002	$x_{6}≻x_{5}≻x_{7}≻x_{8}≻x_{4}≻x_{9}≻x_{3}≻x_{10}≻x_{1}≻x_{2}$
2003	$x_{7}≻x_{5}≻x_{6}≻x_{8}≻x_{4}≻x_{9}≻x_{3}≻x_{10}≻x_{1}≻x_{2}$
2004	$x_{7}≻x_{6}≻x_{5}≻x_{8}≻x_{4}≻x_{9}≻x_{3}≻x_{10}≻x_{1}≻x_{2}$
2005	$x_{7}≻x_{6}≻x_{5}≻x_{8}≻x_{4}≻x_{9}≻x_{10}≻x_{3}≻x_{2}≻x_{1}$
2006	$x_{7}≻x_{6}≻x_{8}≻x_{5}≻x_{4}≻x_{9}≻x_{3}≻x_{10}≻x_{1}≻x_{2}$
2007	$x_{7}≻x_{8}≻x_{6}≻x_{5}≻x_{4}≻x_{9}≻x_{10}≻x_{3}≻x_{1}≻x_{2}$
2008	$x_{7}≻x_{5}≻x_{6}≻x_{8}≻x_{4}≻x_{9}≻x_{10}≻x_{3}≻x_{1}≻x_{2}$
2009	$x_{7}≻x_{6}≻x_{8}≻x_{5}≻x_{9}≻x_{4}≻x_{10}≻x_{3}≻x_{1}≻x_{2}$
2010	$x_{7}≻x_{8}≻x_{6}≻x_{5}≻x_{9}≻x_{10}≻x_{4}≻x_{3}≻x_{1}≻x_{2}$
2011	$x_{7}≻x_{8}≻x_{6}≻x_{5}≻x_{9}≻x_{10}≻x_{4}≻x_{3}≻x_{1}≻x_{2}$
2012	$x_{7}≻x_{8}≻x_{6}≻x_{5}≻x_{9}≻x_{4}≻x_{10}≻x_{3}≻x_{1}≻x_{2}$
2013	$x_{7}≻x_{8}≻x_{6}≻x_{5}≻x_{9}≻x_{4}≻x_{10}≻x_{3}≻x_{1}≻x_{2}$
2014	$x_{7}≻x_{8}≻x_{6}≻x_{5}≻x_{4}≻x_{9}≻x_{10}≻x_{3}≻x_{1}≻x_{2}$
2015	$x_{7}≻x_{8}≻x_{6}≻x_{5}≻x_{9}≻x_{4}≻x_{10}≻x_{3}≻x_{1}≻x_{2}$
2016	$x_{7}≻x_{8}≻x_{6}≻x_{5}≻x_{9}≻x_{4}≻x_{10}≻x_{3}≻x_{1}≻x_{2}$
2017	$x_{8}≻x_{7}≻x_{6}≻x_{5}≻x_{9}≻x_{10}≻x_{4}≻x_{3}≻x_{1}≻x_{2}$
2018	$x_{7}≻x_{8}≻x_{6}≻x_{5}≻x_{9}≻x_{10}≻x_{4}≻x_{3}≻x_{1}≻x_{2}$

**Table A7:** Global stability measures for Spaniards’ preferences attending to several values of λ for Question 2.

	λ				
	**0**	**0.1**	**0.25**	**0.5**	**0.75**
Global preference stability	0.9613445	0.9658650	0.9695323	0.9693923	0.9680178

**Table A8:** Global stability measures for Spaniards’ preferences attending to different regions and several values of λ for Question 2.

	λ				
**Regions**	**0**	**0.1**	**0.25**	**0.5**	**0.75**
Andalucia	0.921849	0.916064	0.901767	0.878874	0.862772
Aragón	0.910084	0.933121	0.957405	0.972920	0.976092
Asturias	0.894118	0.901928	0.909577	0.916134	0.919626
Baleares	0.863866	0.875310	0.888222	0.900539	0.908096
Canarias	0.884874	0.889668	0.896446	0.903622	0.907242
Cantabria	0.865546	0.877306	0.882459	0.872534	0.861259
Castilla la Mancha	0.893277	0.898100	0.901662	0.900201	0.895686
Castilla y León	0.930434	0.940400	0.967679	0.990491	0.997082
Cataluña	0.899160	0.902688	0.904091	0.901932	0.899824
Comunidad Valenciana	0.905882	0.908980	0.914459	0.921917	0.927277
Extremadura	0.875630	0.893600	0.915334	0.932753	0.939525
Galicia	0.884034	0.885832	0.887229	0.886079	0.884161
Madrid	0.931933	0.940310	0.947095	0.951005	0.951675
Murcia	0.873950	0.888473	0.898516	0.899259	0.896087
Navarra	0.905882	0.903787	0.898855	0.893241	0.890410
País Vasco	0.908403	0.906893	0.903737	0.900229	0.899790
La Rioja	0.885714	0.903187	0.922646	0.934991	0.937840

**Table A9:** Global stability measures for Spaniards’ preferences attending to different age ranges and several values of λ for Question 2.

	λ				
	**0**	**0.1**	**0.25**	**0.5**	**0.75**
≤34	0.9479	0.9461	0.9384	0.9269	0.9211
35–54	0.9429	0.9509	0.9581	0.9630	0.9659
≥55	0.9580	0.9598	0.9664	0.9749	0.9777

## References

[1] Kulesher R., Forrestal E. (2014). International models of health systems financing. Int. Model. Health Syst. Financ..

[2] Rodriguez J.A., de Miguel J.M. (1990). The case of Spain. Health Policy.

[3] European Observatory on Health Systems and Policie (2019). Spain: Country Health Profile 2019, State of Health in the EU.

[4] Martinussen P., Magnussen J. (2019). Is having private health insurance associated with less support for public healthcare? Evidence from the Norwegian NHS. Health Policy.

[5] Meleddu M., Pulina M., Scuderi R. (2020). Public and private healthcare services: What drives the choice?. Socio-Econ. Plan. Sci..

[6] Arrow K. (1958). Utilities, Attitudes, Choices: A Review Note. Econometrica.

[7] Gilboa I. (2009). Theory of Decision under Uncertainty.

[8] de Andrés Calle R., Cascón J., González-Arteaga T. (2020). Preferences stability: A measure of preferences changes over time. Decis. Support Syst..

[9] Durán A., Lara J., van Waveren M., Bankauskaite V., WHO (2006). Regional Office for Europe and European Observatory on Health Systems and Policies. Spain: Health System Review.

[10] Arrow K. (1963). Social Choice and Individual Values.

[11] González-Artega T., de Andrés Calle R., Peral M. (2017). Preference stability along time: The time cohesiveness measure. Prog. Artif. Intell..

[12] González-Arteaga T., de Andrés Calle R. New approach to measure preference stability. Proceedings of the 2017 IEEE International Conference on Fuzzy Systems (FUZZ-IEEE).

[13] González-Arteaga T., Cascón J., de Andrés Calle R. (2018). A Proposal to Measure Human Group Behaviour Stability. Information Processing and Management of Uncertainty in Knowledge-Based Systems. Applications, Proceedings of the 17th International Conference, IPMU 2018, Cádiz, Spain, 11–15 June 2018.

[14] González-Arteaga T., Alcantud J., de Andrés Calle R. (2016). A new consensus ranking approach for correlated ordinal information based on Mahalanobis distance. Inf. Sci..

[15] Cascón J., González-Arteaga T., de Andrés Calle R. (2019). Reaching social consensus family budgets: The Spanish case. Omega.

